# Phytochemicals and Obesity: A mini Review from the Dietary Phytochemical Index Perspective

**DOI:** 10.1007/s13668-026-00763-3

**Published:** 2026-04-23

**Authors:** Kadriye Toprak, Zeyneb Yildirim, Dilara Nur Kaplan, Gözde Senturk, Nevin Sanlier

**Affiliations:** 1 Department of Nutrition and Dietetics, School of Health Sciences, Ankara Medipol University, Ankara, Turkey; 2 Department of Nutrition and Dietetics, Institute of Health Sciences, Ankara Medipol University, Ankara, Turkey; 3 Department of Nutrition and Dietetics, Faculty of Health Sciences, Karabuk University, Karabuk, Turkey; 4 Ministry of Youth and Sports, Ankara, Turkey

**Keywords:** Dietary Phytochemical Index, Fat metabolism, Obesity, Phytochemical, Plant-based food, Polyphenol

## Abstract

**Purpose of Review:**

This review aims to evaluate the relationship between the Dietary Phytochemical Index (DPI) and obesity by synthesizing current evidence. While phytochemicals are recognized for their role in preventing chronic diseases, the specific efficacy of the DPI as a predictive metric for obesity management remains a subject of ongoing investigation. This study explores the potential of DPI to serve as a standardized tool for assessing dietary quality in relation to weight control.

**Recent Findings:**

Recent studies retrieved from databases such as Web of Science, Google Scholar, Scopus, and ScienceDirect indicate that phytochemicals exert anti-obesity effects through multiple biological pathways. These include the inhibition of key digestive enzymes like lipase and amylase, appetite regulation, modulation of lipid metabolism, and the suppression of adipocyte differentiation. Current evidence suggests that a higher DPI score is generally associated with improved weight management outcomes; however, results across different populations show some variability that requires careful interpretation.

**Summary:**

Enhancing the dietary intake of phytochemical-rich foods is increasingly recognized as a vital strategy for supporting weight management and promoting overall long-term health. Although the Dietary Phytochemical Index serves as a valuable metric for assessing the quality of phytochemical intake, further comprehensive research is required to establish standardized guidelines and enhance its direct applicability in clinical settings.

**Supplementary Information:**

The online version contains supplementary material available at 10.1007/s13668-026-00763-3.

## Introduction

Phytochemicals are bioactive substances synthesized by plants to defend against many environmental stressors and diseases. While not classified as nutrients, they have demonstrated numerous advantageous benefits for human health due to their biological activities [1–4]. Phytochemicals, which are physiologically active compounds commonly found in plant-based foods such as fruits, vegetables, grains, and legumes, are effective in the prevention and management of non-communicable chronic diseases, including diabetes, cardiovascular diseases, and cancer, owing to their varied activities, especially antioxidant, antimicrobial, and anti-inflammatory properties [5]. Phytochemicals exhibit significant potential in combating obesity through multiple mechanisms and may provide a natural alternative to conventional obesity treatments, which sometimes entail adverse consequences [6]. Phytochemicals, primarily polyphenols, alkaloids, terpenoids, flavonoids, saponins, and steroids, may prevent obesity by regulating carbohydrate and lipid metabolism. Evidence indicates that adherence to a phytochemical-rich diet may help prevent both general and abdominal obesity, particularly among women, as higher phytochemical index scores have been associated with a lower prevalence of obesity [7]. Consistent with these findings, another study reported that phytochemical-rich diets may improve obesity-related outcomes by reducing oxidative stress, modulating proinflammatory cytokine production, enhancing thermogenesis, inhibiting adipocyte differentiation, and decreasing fat cell formation [8].

The Dietary Phytochemical Index (DPI) is a metric utilized to evaluate the phytochemical composition of diets [9]. This score assesses the contribution of phytochemical-rich foods, including legumes, nuts, fruits, vegetables, seeds, and whole grains, to the overall caloric content. Recent research has explored the correlation between dietary phytochemical concentration and obesity [7, 10, 11]. A meta-analysis reported that individuals with high phytochemical index scores had a lower incidence of overweight and obesity, showed a negative correlation between DPI and waist circumference, and indicated that phytochemicals may inhibit preadipocyte proliferation through certain polyphenols and reduce adipogenesis. However, the authors noted that this association appeared more pronounced because the included studies were concentrated in specific geographic regions, and that regional differences in dietary patterns may have influenced the results [12]. In line with these findings, another study emphasized that unhealthy dietary patterns represent the most important modifiable risk factor for overweight and obesity and that inflammatory factors mediate obesity among women with overweight and obesity. The study further reported that adherence to DPI may be associated with improvements in resting metabolic rate (RMR) through reductions in inflammatory markers and may also contribute to obesity treatment, although longer-term studies are required to confirm these effects [13].

Despite the well-documented protective roles of phytochemical-rich foods against chronic diseases, the mechanisms underlying these effects remain only partially understood. Notably, the relationship between the DPI and obesity has not been comprehensively examined in the existing literature. Specifically, it is still uncertain whether DPI, as a separate composite measure, can independently predict obesity outcomes beyond total diet quality and energy intake. Thus, this mini-review aims to critically review existing epidemiological evidence connecting DPI to both general and central obesity, examine possible mechanistic pathways related to habitual dietary consumption, and discuss methodological limitations—such as reverse causality and energy dependence—that hinder the interpretation of these findings.

## Methods

This study was designed as a narrative mini review to synthesize current evidence regarding the relationship between the DPI and obesity. An exploration of literature was carried out by utilizing electronic databases, MEDLINE, Embase, Cochrane Library, CINAHL, ClinicalTrials.gov, Scopus, and Web of Science, covering the period from inception up to January 2026. The reference articles were obtained from databases using the keywords: [Dietary Phytochemical Index], [diet], [obesity], [nutrition], [fat metabolism], [phytochemical], [plant-based food], [polyphenol].

The reviewed studies provided information on age range or mean age, sample size, study setting or location, gender, relevant inclusion and exclusion criteria, and population type (e.g., general population, adults with obesity, children). Details related to DPI methodology were recorded, including the formula used to calculate DPI, which foods were considered phytochemical-rich, the validation status of the dietary assessment tool, and DPI quartiles or mean values by category, when available. Data on obesity measurements (BMI, waist circumference, body weight, etc.), whether general obesity, abdominal obesity, or both were assessed, the DPI–obesity relationship, demographic factors (age, gender, education, etc.), lifestyle factors (physical activity, smoking, etc.), dietary factors (total energy intake, specific nutrients), study type (cross-sectional, longitudinal, case-control, etc.), and study duration or follow-up period, if applicable, were also considered. Finally, the extracted data were qualitatively synthesized to evaluate the consistency of findings across various populations. During the selection process, a total of 145 records were initially identified through database searching. After removing 38 duplicates, the titles and abstracts of the remaining 107 articles were screened. Of these, 48 full-text articles underwent eligibility assessment, resulting in the inclusion of 22 core studies that specifically investigating the association between DPI and obesity outcomes. Additionally, articles written in English were reviewed because the researchers spoke English in addition to their native language. Studies were excluded if phytochemical intake was not analyzed using the dietary phytochemical index, if they were duplicates, or if they were review articles, intervention studies, conference proceedings, letters to the editor, abstracts, or other irrelevant publications. It is important to note that while these 22 clinical and epidemiological studies form the analytical core of this review, the comprehensive bibliography comprises additional supporting literature. These supplementary references were systematically utilized to construct the narrative framework, providing essential context on phytochemical molecular mechanisms, general obesity epidemiology, and regulatory intake guidelines.

Given the narrative nature of this mini review, a formal systematic risk of bias assessment was not conducted; however, each study included in this review was qualitatively evaluated for methodological rigor, focusing on sample size and the adequacy of adjusting for potential confounders (e.g., total energy intake, age, and physical activity) to ensure the reliability of the combined evidence.

### Role of Dietary Phytochemicals in Obesity Management

**Fig. 1:**
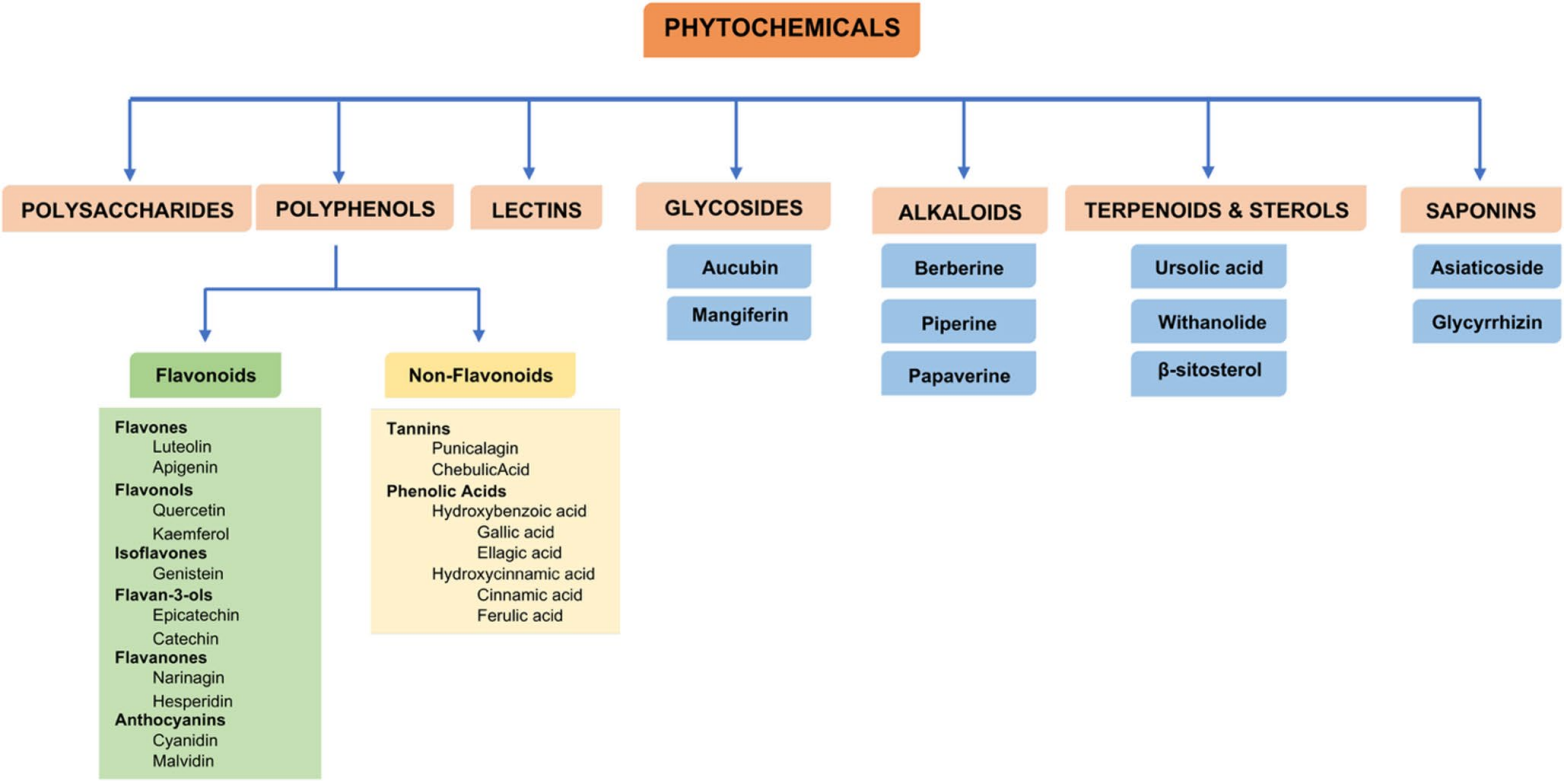
Classification of commonly known phytochemicals [3]

Commonly known phytochemical compounds include phenolic compounds (polyphenols), terpenoids, alkaloids, organosulfides, and nitrogen-containing compounds [2, 3]. Due to their diverse shapes and structures, they have not yet been subjected to a definitive classification [14]. The Fig. 1 shows the classification of commonly known phytochemical compounds [3].

Nutrition is also among the main determinants of obesity and is considered a risk and protective factor. While unhealthy nutrition causes obesity, a nutritional model rich in dietary fiber, phytochemicals, omega-3, and various antioxidant components, as well as the necessary macro and micronutrients, can be effective in preventing the development of obesity [15]. Recent studies strongly support that long-term consumption of plant-based foods (such as grains, vegetables, fruits, legumes, and beverages such as coffee and tea) protects against diseases associated with oxidative stress [16, 17]. In addition, it has been reported that phenolic compounds influence protein synthesis, enzyme activities, gene expression, and protein binding through their interaction with cellular functions at multiple levels [17]. The main approaches to combating obesity include suppressing lipid absorption from the gastrointestinal tract, stimulating fat oxidation, and inhibiting adipogenesis. Phytochemicals are also reported to potentially regulate adipogenesis and adipose tissue, suggesting they effectively prevent obesity [18]. Additionally, evidence suggests that individuals who adhere to a diet rich in phytochemical-containing foods have a reduced incidence of obesity [7, 19].

### Mechanisms of Phytochemicals to Prevent Obesity

Phytochemicals have been shown to reduce adiposity and body fat through multiple pathways, including increasing apoptosis and inhibiting cell proliferation, decreasing lipogenesis and angiogenesis, enhancing lipolysis and insulin sensitivity, and reducing proinflammatory mediators in adipocytes [9, 20]. In addition, higher DPI intake is associated with lower dietary fat intake, greater dietary fiber and protein consumption, and reduced total energy intake. Another explanation for the lower obesity risk associated with high intake of phytochemical-rich foods may relate to their lower glycemic index. It has also been suggested that consuming approximately 40% of total dietary energy from phytochemical-rich foods may protect against weight gain [9, 10, 20]. The literature highlights several biological mechanisms underlying these effects. Phytochemicals positively affect obesity through their important properties, such as modulating lipid metabolism, regulating energy expenditure (thermogenesis), suppressing appetite, controlling fat cell differentiation, and inhibiting lipase activity [21]. However, it is important to connect these mechanistic findings with the concept of the DPI. While the mechanisms described below—often studied using isolated compounds at pharmacological doses—offer biological plausibility, the DPI reflects the habitual consumption of these compounds with an entire diet. Thus, the anti-obesity effects observed in high-DPI diets are likely attributable to the synergistic action of multiple phytochemicals acting at physiological doses rather than a single potent pathway.

***Phytochemicals as Inhibitors of Pancreatic Lipase.*** Phytochemicals have been shown to act on the gastrointestinal tract by inhibiting specific enzymes such as lipase and amylase [22]. Only a few drugs can interact with lipase enzymes and block these enzyme functions. However, although these drugs are clinically approved, they can often lead to gastrointestinal side effects [23]. On the other hand, phytochemicals contained in plant species such as *Platycodi radix*,* Panax japonicas*,* Nelumbo nucifera*, and *Salacia reticulate* are reported to be promising as pancreatic lipase inhibitors. The phytochemicals in this group mainly include tannins, polyphenols, saponins, caffeine, and flavonoids [24]. In studies conducted to find new effective agents, methanol extract obtained from Moricandia arvensis [25]; Ethanol extract of fenugreek leaves [26]; Elite Eriospermum tapos plant has also been confirmed to inhibit pancreatic lipase, α-amylase and α-glucosidase enzymes and exhibit a vigorous antioxidant activity [27].

***Phytochemicals as Energy Expenditure Regulators.*** Proteins have vital roles in regulating energy expenditure processes. For example, uncoupling protein 1 (UCP1) regulates thermogenesis via thyroid hormone and dissipates energy as heat by dissipating the proton gradient generated in oxidative phosphorylation. In addition, UCP3, an analog of UCP1, is known as a potential anti-obesity agent. Many naturally occurring phytochemical compounds such as Capsicum annuum, piperine, caffeine, and resveratrol have been reported to reduce body weight by regulating and increasing UCP expression [24, 28]. At the molecular level, these compounds stimulate energy expenditure primarily through the activation of AMP-activated protein kinase (AMPK) and silent information regulator 1 (SIRT1). Specifically, the SIRT1/PGC-1α signaling pathway acts as a master network for mitochondrial biogenesis, directly upregulating UCP1 transcription and promoting the browning of white adipose tissue [29]. Furthermore, specific phytochemicals like capsaicin activate transient receptor potential vanilloid 1 (TRPV1) channels, triggering calcium-dependent AMPK activation and subsequent lipid oxidation [29, 30]. Fucoxanthin, a natural carotenoid pigment found in brown seaweed, has been shown to support the thermogenesis process in brown adipose tissue [31], while capsaicin stimulates diet-induced thermogenesis and increases the feeling of satiety [32].

***Phytochemicals as Appetite Suppressants.*** Phytochemicals that have been studied and shown to have anti-obesity effects in regulating the hunger and satiety mechanism by reducing appetite include: steroidal glycoside (Hoodia Gordonii and Hoodia Pilifera), hydroxycitric acid, epigallocatechin gallate, saponins (Panax ginseng), EGCG (Camellia sinensis), lectins (Phaseolus vulgaris), HCA (Garcinia cambogia) species. This appetite reduction system is regarded as the initial step in regulating body weight. It involves multifactorial impacts of about 40 anorexigenic and orexigenic hormones, enzymes, and neuropeptides, together with their respective receptors [24, 33, 34]. In the short run, appetite can be modulated by hormonal and neural signals from the gastrointestinal system, which is the largest endocrine organ and is thought to play an important role in controlling appetite by releasing several regulatory peptide hormones. The ghrelin, an orexigenic neuropeptide hormone produced primarily in the stomach, interacts with the growth hormone secretagogue receptor, which is mainly released in the hypothalamus; antagonistic actions mediated by brainstem ghrelin may diminish heightened appetite [33]. Furthermore, at the central nervous system level, specific phytochemicals modulate hypothalamic signaling by stimulating anorexigenic (appetite-suppressing) pro-opiomelanocortin (POMC) neurons while simultaneously suppressing orexigenic (appetite-stimulating) neuropeptide Y/agouti-related peptide (NPY/AgRP) expressions, thereby promoting a sustained feeling of satiety [29, 35]. Some studies suggest that suppressing fatty acid synthase (FAS) activity can reduce both food consumption and body weight [36, 37]. Important phytochemicals such as epigallocatechin gallate in green tea have been shown to help reduce lipid storage and control appetite by inhibiting FAS [38]. In contrast, it has been determined that Capsicum annuum, Camellia sinensis (green tea), and Coffea species do not show consistent results in terms of effectiveness in suppressing the feeling of hunger and satiety [39].

***Phytochemicals as Regulators of Adipocyte Differentiation.*** Some phytochemicals found in plants stand out with their potential to inhibit adipogenesis and have profound regulatory effects on lipid metabolism at the molecular level. Various phytochemicals such as polyphenols found in green tea, vegetables, and fruits such as quercetin and catechin, tannins such as ellagic acid and phytosterols have been reported to reduce adipogenesis [40, 41]. At the transcriptional level, these phytochemicals exert anti-adipogenic effects by downregulating the master regulators of adipocyte differentiation, namely peroxisome proliferator-activated receptor gamma (PPARγ) and CCAAT/enhancer-binding protein alpha (C/EBPα) [29, 42, 43]. Furthermore, specific polyphenols prevent preadipocyte differentiation by activating the Wnt/β-catenin signaling pathway, which actively represses adipogenic gene expression and triglyceride accumulation [42, 43]. In addition, antiadipogenic agents such as resveratrol, lutein, sulfurin, and fisetin have been shown to suppress lipid accumulation and the expression of adipocyte markers in 3T3-L1 and C3H10T1/2 cells, as well as a pair of recently discovered adipogenic genes [44]. Additionally, many natural phytochemicals such as capsaicin, piperine, quercetin, genistein, catechin, and epicatechin have been reported to produce apoptotic effects on maturing fat cells [24].

***Phytochemicals as Lipid Metabolism Regulators.*** It is stated that plant-derived compounds have positive effects on regulating lipid metabolism and controlling obesity. Phytochemicals found in tea and traditional medicinal plants, particularly, stand out in terms of these effects. Caffeine and other compounds dominant in oolong tea have been shown to promote lipolysis by binding to phospholipid phosphate groups and facilitating interactions between lipase and triglyceride elements within lipid droplets [24]. In addition, studies using the aqueous extract (Changkil saponins) of Platycodon grandiflorum, a plant species native to East Asia, have shown that the plant can prevent lipid accumulation by inhibiting pancreatic lipase activity and is associated with lower plasma triacylglycerol concentration [45, 46]. In laboratory studies, phytochemicals from Piper nigrum (black pepper) and Bauhinea pupuria (orchid tree) have been shown to reduce the body weight of obese rats by down-regulating the FAS enzyme [47–49]. Molecularly, this suppression is largely mediated by the inhibition of sterol regulatory element-binding protein 1c (SREBP-1c), the principal transcription factor for de novo lipogenesis, and the inactivation of acetyl-CoA carboxylase (ACC), the rate-limiting enzyme in fatty acid synthesis [29, 42].

***Roles of Phytochemicals in Additional Mechanisms.*** Hormone-sensitive lipase (HSL) is one of the enzymes responsible for releasing free fatty acids from adipose tissue through lipolysis [50]. It has been emphasized that some plant extracts, such as grape seed and Rosemerinus officinalis (rosemary) extract, may have possible inhibitory effects on enzymes such as pancreatic lipase and lipoprotein lipase, especially HSL, and these effects may have a potentially important role in the treatment of obesity [51, 52]. In addition, berberine has been reported to exhibit antimicrobial and anti-obesity activities in the intestine by reducing polysaccharide degradation, increasing fasting-induced fat factor, and regulating related gene expressions [53]. In addition, phytochemicals such as curcumin, genistein, isothiocyanates, and citrus isoflavonoids have been shown to repair epigenetic irregularities in obesity-related genes through epigenetic regulators by acting as natural inhibitors of DNA methyltransferases (DNMTs) and histone deacetylases (HDACs), thereby preventing body weight gain [54]. It is stated that these phytochemicals have antioxidant effects as well as effects on changing epigenetic mechanisms [18].

Table 1 presents the major phytochemical classes and their main anti-obesity mechanisms.

**Table 1:** Major phytochemical classes and main anti-obesity mechanisms

Reference	Some polyphenols, phytochemicals, alkaloids	Main anti-obesity mechanisms
[21, 29, 42, 43], [55– 60]	Polyphenols (quercetin, resveratrol, curcumin, EGCG, anthocyanins, genistein, rutin, fisetin)	↓ adipogenesis/lipogenesis (via PPARγ, C/EBPα and SREBP-1c suppression) ↑ fatty acid oxidation ↑ thermogenesis Antioxidant and anti-inflammatory effects (via NF-κB inhibition, ↓ TNF-α and IL-6) AMPK activation Modulation of Wnt/β-catenin signaling Epigenetic regulation (DNMT/HDAC inhibition) Modulation of gut microbiota (↓ Firmicutes/Bacteroidetes ratio)
[6, 21, 29, 30, 43, 55, 58]	Alkaloids (berberine, capsaicin, caffeine, ephedrine, piperine)	↑ energy expenditure and thermogenesis (via TRPV1/UCP1 upregulation) ↓ appetite (via POMC activation/NPY suppression and ↑ GLP-1 secretion) Increased insulin sensitivity Lipolysis Modulation of Wnt/β-catenin signaling
[29, 56, 58], [61– 63]	Carotenoids, terpenoids, phytoestrogens	Antioxidant and anti-inflammatory effects (via NF-κB inhibition, ↓ TNF-α and IL-6) Regulation of lipid metabolism Adipocyte browning (↑ UCP1 expression) Estrogen receptor-mediated effects in postmenopausal obesity
[35, 42, 43]	Saponins (Panax ginseng, ginsenoside)	↓ appetite (central regulation of POMC, NPY, and AgRP neuropeptides) ↓ adipogenesis (via Wnt/β-catenin activation and PPARγ/C/EBPα suppression) ↓ intestinal lipid absorption and pancreatic lipase inhibition
[42, 54]	Isothiocyanates & Organosulfur Compounds (sulforaphane, allicin)	Epigenetic regulation (HDAC and DNMT inhibition) ↓ adipogenesis/de novo lipogenesis (via PPARγ, C/EBPα, and ACC suppression) Improvement of lipid profile and strong antioxidant defense

EGCG: Epigallocatechin gallate, PPARγ: Peroxisome proliferator-activated receptor gamma, SREBP1: Sterol regulatory element-binding protein 1, NF-κB: Nuclear factor kappa B, TNF-α: Tumor necrosis factor α, IL-6: Interleukin 6, AMPK: Adenosine monophosphate-activated protein kinase, Wnt/β: Wingless-related integration site/β, DNMT: DNA methyltransferases, HDAC: Histone deacetylases, TRPV1: Transient receptor potential vanilloid 1, UCP1: Uncoupling protein 1, POMC: Pro-opiomelanocortin, NPY: Neuropeptide Y, GLP-1: Glucagon-like peptide 1, AgRP: Agouti-related peptide, ACC: Acetyl-CoA carboxylase

Phytochemicals function as metabolic signals involving the PI3K/Akt, AMPK, Wnt/β-catenin, PPARγ/C/EBPα, NF-κB/TLR4, Nrf2, thermogenic (SIRT1–PGC-1α–UCP1), and endocrine pathways. These effects reduce adipogenesis and lipid accumulation, enhance oxidation and thermogenesis, alleviate inflammation and oxidative stress, and stimulate thermogenesis/browning through signaling pathways. This supports the potential use of phytochemicals as multi-targeted anti-obesity agents. Table 2 summarizes the roles of phytochemicals in obesity regarding their major underlying mechanisms and signaling pathways.

**Table 2:** Summary of major mechanisms and signaling pathways of phytochemicals in obesity

Reference	Signaling Pathways	Major Effects on Obesity Biology	Representative Phytochemicals/Sources	Mechanistic Insights
[64–66]	PI3K/Akt	Regulates glucose uptake, lipogenesis, adipogenesis	Anthocyanins, tangeretin (Chenpi), multiple plant metabolites	Regulates GLUT4, FOXO, GSK3β, and mTOR are regulated; components activate PI3K/Akt and PI3K/Akt/GSK3β to reduce lipid accumulation and improve insulin signaling.
[59, 67–69]	AMPK	↑Fatty-acid oxidation ↓Lipogenesis, improves insulin sensitivity, promotes browning	Anthocyanins, EGCG, resveratrol, curcumin, hyperforin	AMPK activation increases mitochondrial biogenesis, inhibits cholesterol and fatty acid synthesis, and improves the processing of hepatic and adipose lipids; hyperforin triggers the AMPK–PGC-1α–UCP1 thermogenic axis.
[43, 70, 71]	Wnt/β‑catenin	Regulator of lipogenesis and adipogenesis	Quercetin, curcumin, resveratrol, ellagic & ferulic acids, many flavonoids	Stabilizes β-catenin, which suppresses PPARγ and C/EBPα to inhibit early adipocyte differentiation and reduce triglyceride accumulation.
[6, 69, 70, 72]	PPARγ / C/EBPα / SREBP1c	Regulator of lipogenesis and adipogenesis	Quercetin, curcumin, resveratrol, ellagic & ferulic acids, many flavonoids	Suppresses adipocyte differentiation by downregulating adipogenic transcription factors; may induce adipocyte apoptosis.
[56, 59, 63, 73, 74]	NF-κB & TLR4	Promotes chronic inflammation in adipose tissue and systemically.	Curcumin, resveratrol, quercetin, other polyphenols	Inhibition of NF-κB/TLR4 reduces TNF-α, IL-6, and MCP-1 levels, decreases macrophage infiltration, and increases insulin sensitivity.
[56, 73]	Nrf2–Keap1	Enhances antioxidant defense and reduces ROS levels.	Isothiocyanates and compounds containing α, β-unsaturated carbonyls (e.g., curcumin)	Activates Nrf2 through covalent interaction with Keap1, upregulating antioxidant enzymes to mitigate ROS-induced adipogenesis.
[68, 69, 73]	SIRT1–PGC‑1α–UCP1 / β3‑AR	Promotes browning of white adipose tissue, thermogenesis, and energy expenditure.	Curcumin, other plant thermogenic compounds	Stimulates co-activation of UCP1, PRDM16, and PPARγ, and mitochondrial biogenesis, it causes the browning of white adipose tissue and increases energy expenditure.
[59, 75]	Adipokines and endocrine signaling	Regulates leptin resistance, adiponectin, and insulin signaling.	Polyphenols, flavonoids, terpenoids, saponins	Improves adipokine profiles and insulin sensitivity by regulating gene-regulatory mechanisms and inhibiting the mTOR/S6K/4EBP pathway.
[6, 59, 72]	Pancreatic lipase / digestive targets	↓ Fat absorption and energy intake	Diverse plant extracts (e.g., anthocyanin-rich fruit juices, herbal preparations)	Direct inhibition of pancreatic lipase and sometimes amylase reduces dietary fat absorption and postprandial lipidemia.

AMPK: Adenosine monophosphate-activated protein kinase, β3-AR: Beta-3 adrenergic receptor, EGCG: Epigallocatechin gallate, FOXO: Forkhead box O, GLUT4: Glucose transporter type 4, GSK3β: Glycogen synthase kinase-3 beta, IL-6: Interleukin-6, MCP-1: Monocyte chemoattractant protein-1, mTOR: Mechanistic target of rapamycin, NF-κB: Nuclear factor kappa B, PGC-1α: Peroxisome proliferator-activated receptor gamma coactivator 1-alpha, PI3K/Akt: Phosphoinositide 3-kinase / Protein kinase B, PPARγ: Peroxisome proliferator-activated receptor gamma, ROS: Reactive oxygen species, SIRT1: Sirtuin 1, SREBP1: Sterol regulatory element-binding protein 1, TLR4: Toll-like receptor, TNF-α: Tumor necrosis factor alpha, UCP: Uncoupling protein

### Phytochemical Index Concept (DPI)

The Dietary Phytochemical Index has been devised as a novel dietary index for assessing the benefits of phytochemically rich food intake through population-based epidemiological studies [9]. Given the numerous health benefits associated with phytochemicals, measuring the amount of phytochemicals in the diet is essential. However, it is not easy to estimate the dietary phytochemical composition or human tissue sample [76].

Therefore, the DPI emerges as a significant tool for estimating the phytochemical content of the diet. McCarty (2004) defines the Dietary Phytochemical Index as the proportion of dietary energy obtained from phytochemical-rich foods [77]. The index is calculated using the formula below:

$$DPI=\frac{Daily\:energy\:intake\:derived\:from\:phytochemical\:rich\:foods\:(kcal)}{Total\:daily\:energy\:intake\:(kcal)}\times100$$

DPI is calculated based on the energy content of consumed foods. For instance, the ratio of energy derived from phytochemical-rich foods, such as vegetables, fruits, whole grains, and oilseeds, to the total energy intake determines this value. However, there are certain limitations in obtaining the DPI. Foods rich in phytochemicals but lacking energy, such as spices, tea, and coffee, are excluded from the calculation. This exclusion affects DPI scores and is considered a limiting factor [12].

Due to the effectiveness of phytochemicals in preventing and treating chronic diseases, the relationship between phytochemicals and diseases has become a significant research topic in recent years. In this context, promising studies examine the relationship between DPI and disease. Observational research has assessed the relationship between chronic diseases and DPI, including stroke [55], hypertension [78], cancer [79], obesity [10], osteoporosis [80]. In recent years, the DPI has been increasingly utilized as a significant research tool in obesity-related studies. This review focuses on the potential relationship between DPI and obesity and critically examines the existing literature in this field.

### Studies on the Relationship Between Phytochemical Index and Obesity

Although it is well known that increased consumption of phytochemical-rich foods is associated with favorable outcomes in obesity [41, 44], the number of studies examining the relationship between obesity and DPI in the literature remains limited. Table 3 summarizes cross-sectional and longitudinal studies available in the literature, and this section presents a compiled review of these studies.

**Table 3:** Studies related to Phytochemical Index and obesity

Reference	Study Design and Population	Method	Finding Type	Key Results
[7]	Cross-sectional; *n* = 57,940 adults	Demographic and Lifestyle Data, Anthropometric assessments, 24-hr Dietary Recall questionnaires, DPI	Positive (Women) / Null (Men)	Among participants, women tended to have a higher DPI than men. Higher DPI linked to significantly lower abdominal obesity in women; no significant relation in men.
[10]	Cross-sectional; 356 children	Anthropometric assessments, FFQ, DPI	Positive	Highest DPI quartile had notably reduced odds of being overweight/obese.
[11]	Cross-sectional; *n* = 2567 adults	Clinical assessment, Lifestyle assessments, Biological measurements, FFQ, DPI	Positive	66% lower risk of abdominal obesity and 36% lower risk of hypertriglyceridemia in higher DPI quartiles.
[13]	Cross-sectional; *n* = 404 women	Sociodemographic data, Anthropometric assessment, IPAQ, Biochemical assessments, FFQ, DPI	Positive	Compliance with a diet high in phytochemical content has been shown to reduce the risk of hypometabolism by lowering inflammatory mediators such as MCP-1, PAI-1, and TGF-β.
[19]	Cross-sectional (KNHANES); *n* = 31,319 adults	Sociodemographic data, Anthropometric assessments, 24-hr Dietary Recall questionnaires, DPI	Positive	Those in the highest DPI quintile showed significantly lower rates of abdominal obesity (OR: 0.90, 95% CI: 0.81–0.99).
[20]	Case-Control; *n* = 300 adults	Sociodemographic data, Anthropometric assessment, IPAQ, Biochemical assessments, FFQ, DPI	Positive	DPI was found to be inversely associated with fasting blood glucose and OGTT values. OR for prediabetes significantly decreased across increasing DPI quartiles (*P* < 0.001).
[81]	Cross-sectional; *n* = 844 adults	Sociodemographic data, Physical activity, Anthropometric assessment, FFQ, DPI	Mixed / Null	No association between DPI and general obesity. Women in the top quartile of the DPI had a lower odd of central obesity by WC. Men in the third quartile of the DPI were at lower risk of central obesity by waist-to-hip ratio.
[82]	Cross-sectional; *n* = 850 adults	Anthropometric and blood pressure assessments, Physical activity, FFQ, DPI	Positive (Women) / Null (Men)	The mean score of DPI in women and men was 36.2 ± 26.8 and 33.7 ± 24.7, respectively. An inverse association between DPI and the risk of abdominal obesity was identified in women (ORfourth vs. first quartile, 0.54; 95% CI, 0.29–1.00; p trend = 0.03).
[83]	Cross-sectional (Colaus); *n* = 3879 adults	Demographic and Lifestyle Data, Anthropometric assessments, biochemical parameters, FFQ, DPI	Positive	Notable inverse associations were identified between the two highest quartiles of DPI and WC, BMI, insulin, leptin, and hs-CRP.
[84]	Longitudinal (3-year); *n* = 1938 adults	Anthropometric assessment, FFQ, DPI	Positive	Throughout a three-year observational period, it was found that an elevated energy intake exceeding 37%, derived from phytochemical-rich sources, could potentially mitigate weight gain and reduce adiposity in adult individuals.
[85]	Cross-sectional; *n* = 651 old men	Anthropometric assessment, 24-hr Dietary Recall questionnaires, Net Endogenous Acid Production, DII, DPI	Positive	Diet may have a role in the development of obesity through inflammatory modulation mechanisms in the elderly. There is a significant negative correlation between DII and DPI scores
[86]	Cross-sectional; *n* = 228 overweight and obese women	Body composition analysis (with BIA), biochemical parameters, FFQ, DPI	Positive	After controlling for potential confounding factors, women in the highest tertile of DPI were significantly less likely to have a metabolically unhealthy overweight/obesity phenotype than those in the lowest tertile.
[87]	Cross-sectional (KNHANES); *n* = 1196 preschoolers	Sociodemographic data, Anthropometric assessments, 24-hr Dietary Recall questionnaires, DPI	Positive	The prevalence of obesity was significantly lower in boys and higher DPI group. The participant’s BMI, weight, and total energy intake did not differ significantly.
[88]	Cross-sectional; *n* = 10,000 adults	Demographic and Lifestyle Data, Anthropometric assessments, DPI, FFQ, IPAQ, Laboratory measurements	Positive	Participants in the second and fourth DPI quartiles, showed a 30% and 25% lower risk of abdominal obesity, respectively. Women in the highest DPI quartile had a 59% lower risk of MetS compared to those in the lowest quartile.
[89]	Cross-sectional; *n* = 639 women	Sociodemographic and lifestyle Data, Anthropometric assessments, 24-hr Dietary Recall questionnaires, DPI, BCR, BCKL	Null	DPI was not associated with WC, BMI, BCR, or BCKL. BMI was significantly correlated with WC and BCR (*r* = 0.719 and *r* = 0.605, respectively).
[90]	Case-control; *n* = 331 adults	Sociodemographic data, Anthropometric assessment, blood pressure assessments, 24-hr Dietary Recall questionnaires, DPI	Null (Index) / Positive (Polyphenols)	DPI score itself was not associated with BMI; however, total polyphenol intake was inversely related to BMI and WC.
[91]	Cross-sectional; *n* = 404 women	Sociodemographic data, Anthropometric assessment, IPAQ, Biochemical assessments, FFQ, DPI	Positive	Intakes of stilbenes and lignans were inversely associated with BMI (*P* = 0.04, *P* = 0.02). Polyphenol intake showed an inverse association with waist-to-hip ratio (*P* = 0.04). Stilbenes were linked to lower cholesterol (*P* = 0.03), other polyphenols to lower triglycerides (*P* = 0.01), and lignans to reduced HOMA-IR (*P* = 0.03).
[92]	Cross-sectional; *n* = 4296 children	Anthropometric assessment, Physical activity, FFQ, DPI	Null (Statistical)	Higher DPI associated with lower risk of obesity, but findings were not statistically significant. Higher phytochemical intake had more favorable anthropometric profiles.
[93]	Cross-sectional; *n* = 54 overweight young adults	Sociodemographic data, Anthropometric assessment, Biochemical assessments, FFQ, DPI	Positive	DPI values were higher in the overweight and obese group. Significant correlations were found between the DPI score and BMI, WC, waist-to-hip ratio, and plasma oxidative stress.
[94]	Cross-sectional; *n* = 203 adolescents	Anthropometric assessment, Biochemical assessments, PAQ-A, FFQ, DPI	Positive	Higher DPI significantly associated with a healthier metabolic profile in overweight/obese individuals.

BCR: breast cancer risk, BCKL: breast cancer knowledge level, BIA: bioelectrical impedance analyzer, BMI: Body Mass Index, DII: Diet Inflammatory Index, DPI: Dietary Phytochemical Index, FFQ: Food Frequency Questionnaire, IPAQ: International Physical Activity Questionnaire, Metabolic Syndrome (MetS), OGTT: Oral glucose tolerance test, PAQ-A: Physical activity questionnaire for adolescents, WC: Waist Circumference

According to Asgari et al. (2021), no significant correlation was found between DPI and general obesity among adults after adjusting for age, gender, and energy intake [81]. However, even after these adjustments a negative relationship was found between central obesity and DPI scores, especially among females. This suggests that while the link between DPI and total weight may be affected by energy balance, its possible connection to abdominal fat distribution might be more independent [81]. Mechanistically, Asgari et al. [81] and Im et al. [7] suggest that this gender-specific link may be further explained by the structural similarity between certain phytochemicals, such as isoflavones and lignans, and endogenous estrogens. These phytoestrogens can mimic or influence estrogenic signaling, which is crucial in regulating adipose tissue distribution and metabolic homeostasis, especially in women [7, 81]. Also, there are studies supporting the relationship between DPI and central obesity [88, 89]. A three-year follow-up study revealed that individuals consuming phytochemical-rich foods (especially whole grains, fruits, and oilseeds) was associated with a lower likelihood of weight gain and favorable changes in body fat by increasing the DPI score. Notably, although higher DPI was associated with lower total energy and fat intake and higher dietary fiber consumption at baseline, the inverse association between DPI and 3-year changes in weight and adiposity remained significant after adjusting for these potential confounding variables [84]. Additionally, based on the study by Gamba et al. (2023), high DPI has been inversely associated with weight gain and waist circumference [83]. The authors suggested that these positive effects could be linked to the high dietary fiber content and lower energy density naturally found in phytochemical-rich diets, although the protective role of phytochemicals themselves through antioxidant pathways remains a key factor [83]. Additionally, Gamba et al. [83] observed that these protective effects were especially noticeable in women. This could be attributed to the interaction between phytochemicals and female sex hormones, as well as the modulation of adipokines. Since women typically have higher circulating leptin levels than men, they may be more responsive to the leptin-sensitizing and anti-inflammatory effects of phytochemical-rich diets, which can lead to benficial changes in waist circumference and body fat [83]. In another study investigating the association of diet on the development of inflammation-related conditions such as obesity, a steady decrease in the Dietary Inflammatory Index (DII) score was observed with increasing DPI [85]. The DII assesses overall diet quality based on its inflammatory potential, and the observed negative relationship between DII and DPI indicates that a phytochemical-rich diet might be linked to reduced obesity-related systemic inflammation. Importantly, in this study, participants with the lowest DII scores (more anti-inflammatory) also exhibited significantly lower BMI, waist circumference, and body fat percentage, further reinforcing the connection between dietary anti-inflammatory capacity and weight control [85].

In a study involving women with overweight/obesity, DPI has been found to have an inverse relationship with HOMA-IR and triglyceride levels in individuals with metabolically unhealthy obesity [86]. This indicates that DPI may serve as a marker for metabolic health regardless of total body weight. A community-based study conducted in Korea found that women generally had higher DPI scores than men; specifically, women in the highest DPI category had lower prevalences of obesity and abdominal obesity [7]. These associations remained significant even after adjusting for total energy intake and physical activity, although no such relationship was observed among male participants for general obesity [7]. Similarly, in the study by Kim et al. (2020), women were found to have higher DPI scores compared to men [19]. Beyond biological factors, this may reflect behavioral differences, as women often exhibit higher health consciousness and a greater tendency to adopt plant-based dietary patterns as a proactive weight-management strategy [19]. Likewise, there are studies indicating that as DPI increases, the risk of abdominal obesity decreases [11, 19]. A study evaluated the use of the DPI in the pediatric population. A study conducted in South Korea with preschool children showed that a higher DPI score was associated with a lower prevalence of obesity, particularly in boys [87]. Notably, this association remained significant after adjusting for age and total energy intake, suggesting that the phytochemical density of the diet-rather than just total caloric consumption-may be contributing factor in weight management during early childhood development [87]. Similarly, a study from Iran reported an inverse relationship between higher DPI and the risk of overweight and obesity in children. A higher DPI was also associated with enhanced diet quality, defined by a higher potassium, dietary fiber, and vitamin C intake among the study group. Notably, the association between higher DPI and lower odds of overweight/obesity remained significant after adjusting for age, sex, energy intake, and physical activity, although the researchers noted that the naturally high fiber content of phytochemical-rich diets is an important characteristic to consider when evaluating these metabolic benefits [10].

The relationship between DPI and MetS has been examined in various studies. In a cross-sectional analysis conducted by Gamba et al. (2023) involving 3879 participants, no significant association was observed for MetS or its components except for central obesity [83]. Similarly, in a study involving 850 participants aged 18–65 conducted by Firouzabadi et al. (2021), a reverse relationship was found between DPI and increased central obesity risk in women [82]. Moreover, Vasmehjani et al. (2021) evaluated in their study that women in the highest quartile of DPI had a 59% lower risk of MetS compared to women in the lowest quartile of DPI [88]. As far as we know, only one meta-analysis study by Wei et al. (2022) examines the relationship between DPI and obesity [12]. The meta-analysis found that the studies examined were cross-sectional studies with high heterogeneity in terms of gender and region. The results of the meta-analysis study show that a high DPI score is associated with a reduced risk of overweight/obesity. However, the authors noted that phytochemical-rich diets are often lower in calories and that high heterogeneity among studies may arise from different dietary patterns and varying adjustments for lifestyle factors across regions [12].

A comparative evaluation of the reported effect sizes shows a high level of heterogeneity in the strength of associations across various study designs. Meta-analytical evidence [12] consistently indicates more robust and statistically significant effect sizes (OR: 0.81), whereas individual cross-sectional studies—such as those by Asgari et al. [81] and Kim et al. [19]—present more varied results, often demonstrating stronger associations specifically for central obesity rather than general BMI. Additionally, longitudinal data [84] reveal that while the annual weight gain prevention is modest in absolute terms, the cumulative long-term impact on adiposity remains clinically significant. The strength of these associations appears to be significantly influenced by the level of adjustment for dietary fiber and energy density; studies that isolated the independent effect of phytochemicals through more stringent multivariable models often reported smaller, yet more precise, effect sizes.

It is also essential to carefully consider the possible overlap between the DPI and other dietary quality indices, such as the Mediterranean Diet Score (MDS) and the Dietary Inflammatory Index (DII). Although these indices share key conceptual and statistical similarities, they are not exactly the same. For example, studies have demonstrated a strong inverse relationship between the DPI and the DII [85], indicating that a high-DPI diet tends to be anti-inflammatory. However, while the MDS and DII incorporate broader nutritional factors like animal-derived nutrients and specific fat ratios, the DPI uniquely focuses on the caloric content of phytochemical-rich foods. Therefore, the DPI should be regarded as a complementary measure that specifically highlights the phytochemical-driven advantages of a high-quality diet.

When analyzing the combined evidence presented in these studies, it is necessary to addressed two important methodological challenges, namely reverse causality and residual confounders. Since most of the reviewed literature, including the meta-analysis by Wei et al. [12], uses cross-sectional designs, the direction of the association remains complex. It is plausible that individuals with a higher body mass index (BMI) or existing obesity may have changed their dietary habits toward fewer phytochemical-rich foods due to preexisting metabolic conditions or weight-related lifestyle shifts, rather than a low DPI being the main cause of weight gain. Additionally, although many studies adjusted for primary confounders like age, sex, and total energy intake, the risk of residual confounding persists. Unmeasured factors such as psychological stress, sleep hygiene, and nuanced socioeconomic behaviors—which often cluster with both dietary choices and metabolic health—could potentially influence the observed DPI–obesity associations. Therefore, while the current findings provide a consistent signal, they should be interpreted with caution until confirmed by longitudinal designs that can better decouple these temporal and lifestyle-related interactions.

Another striking finding emerging from the epidemiological evidence summarized in Table 3 is that, although a high Dietary Phytochemical Index (DPI) does not always lead to a consistent reduction in overall Body Mass Index (BMI) after energy adjustment, it shows a strong inverse relationship with waist circumference and abdominal obesity (81, 90). While BMI reflects total body mass, including muscle and water, waist circumference specifically represents visceral adiposity. This suggests that phytochemicals possess a specific underlying physiological mechanism. At the metabolic level, phytochemicals stimulate lipid oxidation and suppress de novo lipogenesis by activating the AMPK and SIRT1 pathways, particularly in the more metabolically active visceral adipose tissue [29, 42, 43]. In addition, since visceral adiposity is directly characterized by chronic low-grade inflammation and macrophage infiltration, the potent anti-inflammatory properties of phytochemicals play a specific role in reducing visceral fat. Phytochemicals suppress the local release of pro-inflammatory cytokines, such as TNF-α and IL-6, in visceral adipose tissue by inhibiting the NF-κB signaling pathway. This anti-inflammatory effect, when combined with epigenetic regulatory mechanisms (such as DNMT/HDAC inhibition), specifically attenuates visceral adipocyte hypertrophy [29, 43, 54]. Therefore, a high DPI can preserve lean muscle mass and promote favorable fat redistribution; this mechanistically explains why a significant improvement is observed in central obesity (waist circumference) even in the absence of profound changes in overall body weight.

### Safe Intake Levels of Phytochemicals

The European Food Safety Authority (EFSA) has not established specific daily intake recommendations for phytochemicals targeting obesity prevention and weight management [95]. However, it is reported that safety assessments by EFSA are necessary, and that establishing harmonized regulatory definitions or recommended intake levels for most phytochemicals would be beneficial [96]. Current research suggests emphasizing the consumption of phytochemical-rich foods (fruits, vegetables, whole grains, legumes, nuts) and using the “phytochemical index” (defined as the percentage of dietary energy derived from plant foods) rather than specific mg/day intake targets [7, 87, 90]. Consistent with this food-based approach, major guidelines such as the Dietary Guidelines for Americans and the World Health Organization (WHO) generally recommend a minimum total intake of 400 g/day (approx. 5 servings) of fruits and vegetables [97–99]. This highlights the specific value of the DPI. Since exact daily targets for phytochemicals are not yet defined, the DPI offers a more precise metric by evaluating the quality of calories (energy percentage) derived from these foods, rather than relying solely on the weight of food eaten [7, 8, 98, 100, 101].

## Conclusion

Phytochemicals are plant-derived compounds that exhibit antioxidant, anti-inflammatory, anti-adipogenic, and lipolytic properties in the body. Recent studies indicate that increased intake of a phytochemical-rich diet is advantageous for obesity and abdominal obesity. Phytochemicals prevent obesity through various mechanisms, including the inhibition of specific enzymes like lipase and amylase, regulation of hunger and satiety by appetite suppression, modulation of lipid metabolism, control of obesity, inhibition of adipogenesis, and regulatory effects on fat metabolism. Improving individuals’ dietary choices and increasing energy intake from foods rich in phytochemicals may positively affect weight management and enhance health metrics.

The dietary phytochemical index provides critical standards for evaluating the amounts of dietary phytochemicals. Applying DPI is increasingly vital for understanding the potential effects of phytochemicals on health and clarifying their relationship with chronic diseases, including obesity. While numerous pathways concerning the link between obesity and phytochemicals are documented in the literature, there is a paucity of studies investigating the association between obesity and DPI. Numerous studies have evaluated substantial correlations between DPI and obesity. Nonetheless, some studies indicate the opposite.

In conclusion, although several limitations exist regarding the application of the DPI, increasing phytochemical intake through the diet remains a promising strategy in the context of obesity, and the DPI serves as a valuable tool for evaluating this intake. Current evidence suggests that while DPI is strongly associated with favorable obesity outcomes, further research is needed to confirm whether it predicts obesity risk independently of overall diet quality and energy intake.

### Suggestions and Future Perspective

DPI differs from rapid screening tools based on portion counting (e.g., Mediterranean Diet Adherence Scale - MEDAS) in terms of clinical usefulness. MEDAS is practical and quick; however, DPI offers a deeper metabolic view in obesity management because it measures not only the amount but also the ‘energy quality’ (phytochemical density) of the diet. Still, DPI calculations usually require nutrient intake records (such as semi-quantitative FFQs) and nutrient analysis software, making it less of a quick and immediate tool for busy clinical settings. Nevertheless, in detailed dietetic assessments, it provides a valuable metric for shifting the focus from simply ‘calorie restriction’ to ‘improving the source of calories(5a).

Beyond its use in clinical settings, DPI also has significant potential for shaping public health policies and obesity prevention strategies. Unlike traditional guidelines that emphasize absolute portion sizes (e.g., 5 servings per day), integrating DPI into public health initiatives could shift the focus to ‘phytochemical intensity’. Setting specific DPI thresholds for institutional meal menus, such as in schools or workplaces, can help ensure meals have a minimum level of phytochemicals relative to their calorie content. Moreover, obesity prevention guidelines could set DPI scores as clear behavioral goals. This approach could be an effective way to combat the obesogenic environment by encouraging the consumption of plant-based foods instead of high-calorie processed options.

Consumption of phytochemical-rich foods and higher DPI levels appears to be associated with a lower risk of obesity-related conditions, including metabolic syndrome, cardiovascular disease (CVD), hypertension, and cancer. However, because the number of publications is limited and clinical evidence remains insufficient, large-scale prospective cohort studies and randomized clinical trials are needed to substantiate these associations.

Further research is also needed to refine the DPI and facilitate its broader application in clinical and public health settings. In particular, longitudinal and intervention studies should aim to improve the DPI so that it more accurately reflects phytochemical content independent of total energy intake. Future work should also assess the value of the DPI as an effective tool for evaluating and monitoring population-level obesity prevention efforts. Future studies should also focus more on human populations and carefully account for potential confounding factors.

Food–drug interactions are clinically important because they can alter the efficacy of disease treatment and increase the risk of toxicity. Certain substances present in polyphenol-rich foods may modify the activity of enzymes and transporters involved in intestinal drug metabolism. Therefore, it is advisable for future research to take potential food–drug interactions into account.

### Limitations

Evidence indicates that higher dietary phytochemical intake is associated with a lower risk of obesity, particularly abdominal obesity. However, a key limitation of this review is the inherent methodological nature of the primary literature. To date, the relationship between DPI and obesity has been studied almost entirely through cross-sectional approaches, which are useful for identifying associations but cannot establish temporal precedence or causality. The noticeable absence of randomized controlled trials (RCTs) specifically examining DPI remains a major barrier to translating these findings into clear clinical guidelines. Furthermore, the predominance of cross-sectional study designs and the geographic concentration of available research limit causal inference and restrict the generalizability of findings to broader populations. Moreover, considerable heterogeneity exists in the methods used to assess nutritional status across studies.

Finally, in addition to the limitations of the study design, there are also methodological constraints associated with the Dietary Phytochemical Index (DPI). Because DPI is calculated based on the percentage of energy derived from phytochemical-rich foods, it primarily reflects calorie-dense plant sources such as fruits and grains, while potentially underestimating the contribution of phytochemical-rich but calorie-free (or low-calorie) sources such as tea, coffee, spices, and herbs. Consequently, DPI may function more as a proxy for overall dietary quality or fibre intake rather than as a precise measure of total phytochemical exposure. Furthermore, the current calculation does not consider the bioavailability or potency of specific phytochemical subclasses. Future research should aim to integrate these calorie-free sources and bioavailability factors into phytochemical indices to improve exposure assessment.

## Key References

- Vasmehjani, A. A., Darabi, Z., Nadjarzadeh, A., Mirzaei, M., & Hosseinzadeh, M. (2021). The relation between dietary phytochemical index and metabolic syndrome and its components in a large sample of Iranian adults: a population-based study. BMC public health, 21(1), 1587. https://doi.org/10.1186/s12889-021-11590-2
  - ○ With its massive sample size of 10,000 individuals, this landmark study provides robust statistical evidence that high phytochemical intake can reduce the risk of metabolic syndrome by 59%, particularly in women.
- Kim M, Park K. Association between phytochemical index and metabolic syndrome. Nutr Res Pract. 2020; https://doi.org/10.4162/nrp.2020.14.3.252
  - ○ This study, with its large sample size of over 31,000 participants, is one of the most comprehensive recent studies demonstrating that a high phytochemical index (DPI) significantly reduces the prevalence of abdominal obesity, hyperglycaemia, and metabolic syndrome.
- Gamba M, Roa-Diaz ZM, Raguindin PF, Glisic M, Bano A, Muka T, et al. Association between dietary phytochemical index, cardiometabolic risk factors and metabolic syndrome in Switzerland: the CoLaus study. Nutr Metab Cardiovasc Dis. 2023; https://doi.org/10.1016/j.numecd.2023.07.018
  - ○ Based on cohort data, this study is critical in that it demonstrates the inverse relationship between high phytochemical intake and not only body composition but also inflammatory markers such as insulin, leptin, and hs-CRP.
- Han, Y. J., Baek, J. H., Jung, S. K., Yang, J. S., Shin, N. R., & Park, M. Y. (2023). Association between the Dietary Phytochemical Index and Lower Prevalence of Obesity in Korean Preschoolers. Nutrients, 15(11), 2439. https://doi.org/10.3390/nu15112439
  - ○ This study, which fills a gap in the DPI literature, is a rare and unique paediatric study showing that a diet high in phytochemical content reduces the risk of obesity in preschool children.

## Supplementary Information

Below is the link to the electronic supplementary material.

Supplementary Material 1

Supplementary Material 2

## Data Availability

All data needed to evaluate the conclusions in this article are included in the article. Additional data related to this article may be requested from the corresponding author.

## Abbreviations

BMI: Body Mass Index
CVD: cardiovascular disease
DII: Dietary Inflammatory Index
DPI: Dietary Phytochemical Index
EFSA: European Food Safety Authority
FAS: fatty acid synthase
HSL: Hormone-sensitive lipase
RMR: resting metabolic rate
UCP: uncoupling protein
WHO: World Health Organization
