# Supplementary figures and images for: Phytochemicals and Obesity: A mini Review from the Dietary Phytochemical Index Perspective
Source: Curr Nutr Rep. 2026 Apr 23;15(1):37. doi: 10.1007/s13668-026-00763-3 (PMC13102861; doi:10.1007/s13668-026-00763-3)

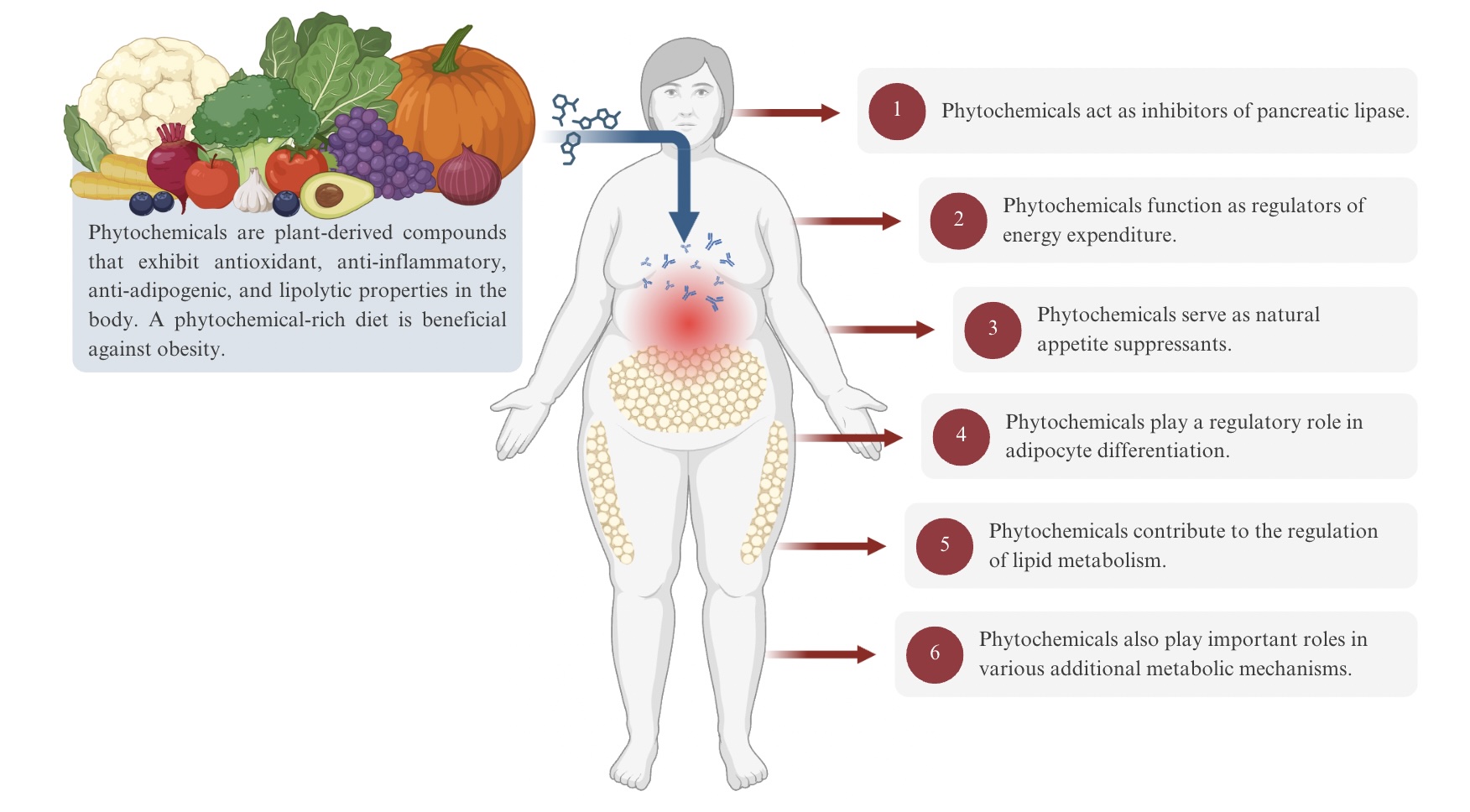

Supplement: Supplementary file 1 — Supplementary Material 1 [file 13668_2026_763_MOESM1_ESM.jpg]

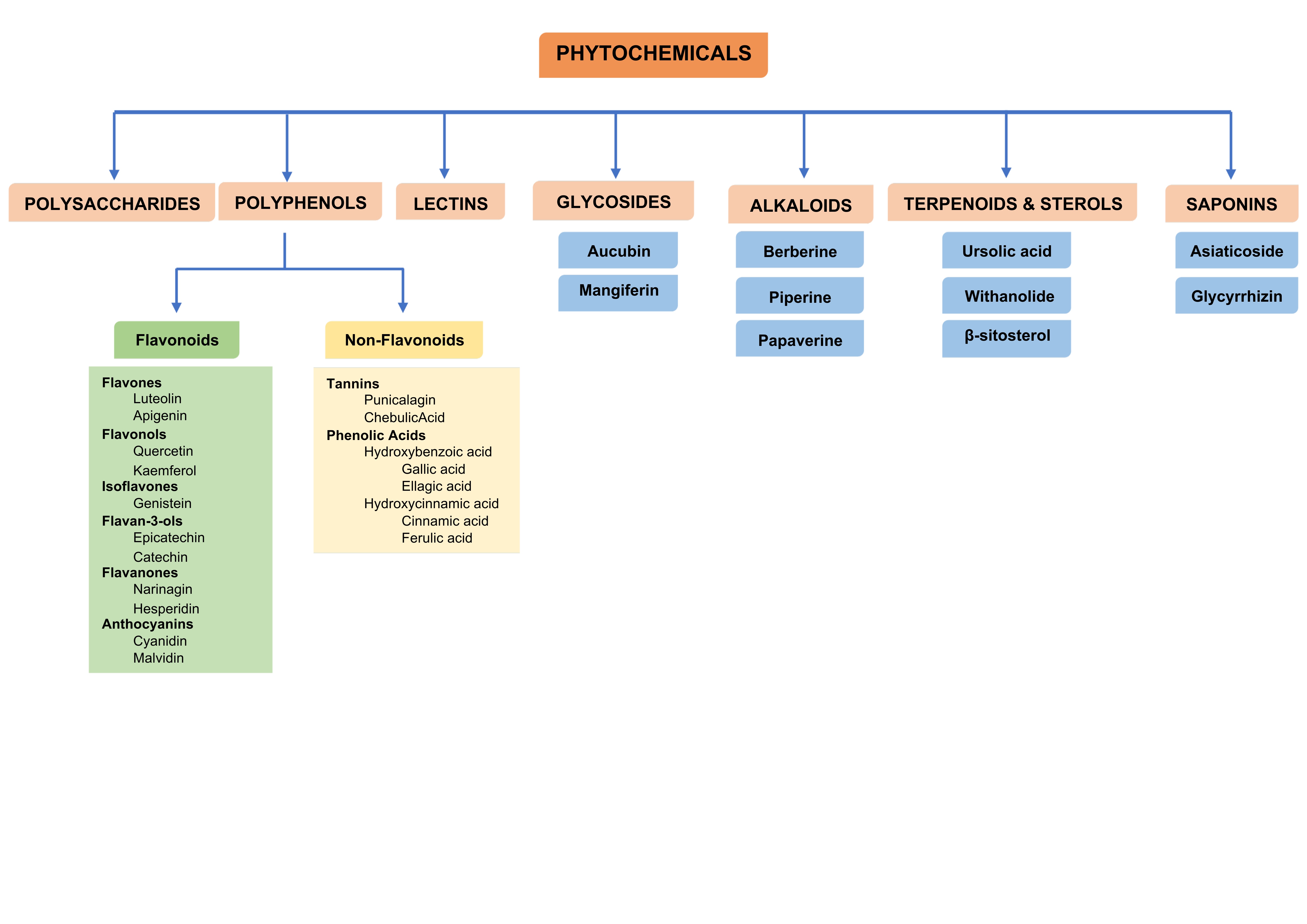

Supplement: Supplementary file 2 — Supplementary Material 2 [file 13668_2026_763_MOESM2_ESM.jpg]
